# Influences of copper–potassium ion exchange process on the optical bandgaps and spectroscopic properties of Cr^3+^/Yb^3+^ co-doped in lanthanum aluminosilicate glasses

**DOI:** 10.1039/d0ra10831f

**Published:** 2021-02-26

**Authors:** T. H. Le, Anh-Luan Phan, Nguyen Minh Ty, Dacheng Zhou, Jianbei Qiu, Ho Kim Dan

**Affiliations:** Department of Physics and Technology, Thai Nguyen University of Sciences Thai Nguyen Vietnam; Institute of Fundamental and Applied Sciences, Duy Tan University Ho Chi Minh City 700000 Vietnam phananhluan@duytan.edu.vn; Faculty of Natural Sciences, Duy Tan University Da Nang City 550000 Vietnam; Faculty of Natural Sciences, Thu Dau Mot University Thu Dau Mot 590000 Vietnam; Key Laboratory of Advanced Materials of Yunnan Province, School of Materials Science and Engineering, Kunming University of Science and Technology Kunming 650093 China; Ceramics and Biomaterials Research Group, Advanced Institute of Materials Science, Ton Duc Thang University Ho Chi Minh City Vietnam hokimdan@tdtu.edu.vn; Faculty of Applied Sciences, Ton Duc Thang University Ho Chi Minh City Vietnam

## Abstract

In this study, lanthanum aluminosilicate glasses with compositions of 45SiO_2_–20Al_2_O_3_–12.5LaF_3_–10BaF_2_–9K_2_O–1Cr_2_O_3_–2.5Yb_2_O_3_ (SALBK) were prepared using the conventional melting method and copper–potassium ion exchange process. Influences of the ion exchange process between copper and potassium on the visible, upconversion, and near-infrared luminescence spectra of Cr^3+^/Yb^3+^ co-doped under excitations of 343, 490, and 980 nm LD were investigated. The EDS analysis of SALBK glasses was measured to confirm the presence of atoms in the glasses. The values of direct and indirect bandgaps of Cr^3+^/Yb^3+^ co-doped SALBK glasses were calculated and analyzed. Besides, the energy exchange processes between Cu^+^, Cu^2+^ ions, and Cr^3+^, Yb^3+^ ions were also proposed and discussed.

## Abstract 2

Visible emission spectra of SALBK-1Cr2.5Yb-0Cu, SALBK-1Cr2.5Yb-15Cu, SALBK-1Cr2.5Yb-20Cu, SALBK-1Cr2.5Yb-25Cu, SALBK-1Cr2.5Yb-30Cu, and SALBK-1Cr2.5Yb-35Cu glass samples under excitation 343 nm.

## Graphical abstract

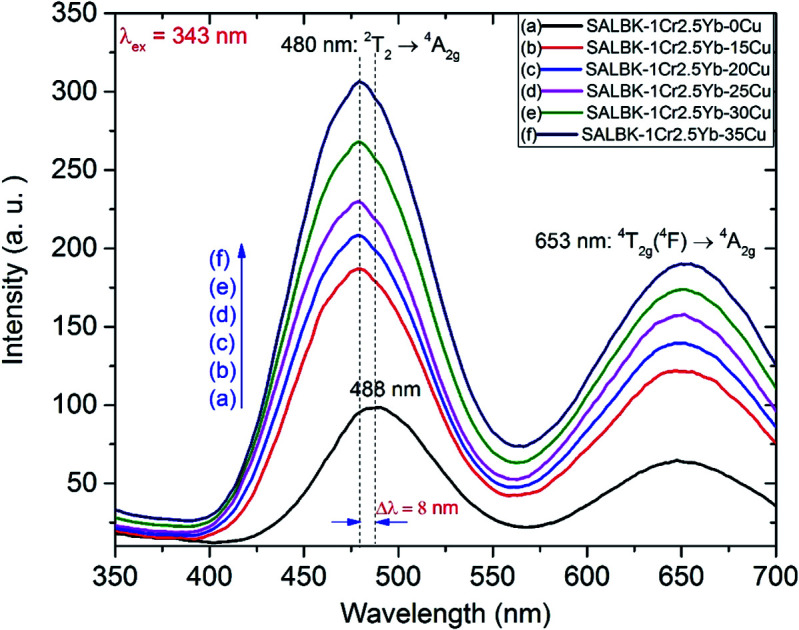

## 1. Introduction

In recent years, the spectroscopy and optical properties of chromium single-doped and chromium/rare-earth (RE) co-doped have been extensively studied^1–5^ due to their advantages.^6,7^ Chromium is a transition metal (TM) with many different valence states.^8–10^ In the glass networks, it often exists in the trivalent state Cr^3+^,^1–5,8^ which can emit radiation in the visible (VIS), near-infrared (NIR) regions under different excitation wavelengths.^5,11,12^ Also, the ^4^T_1g_(F) → ^4^A_2g_, ^4^T_2g_(F) → ^4^A_2g_, ^2^T_1g_ → ^4^A_2g_, ^2^T_2g_ → ^4^A_2g_ and ^2^E → ^4^A_2g_ transitions of Cr^3+^ can be combined with Yb^3+^ to generate the VIS, NIR emission spectra.^5,13,14^ In 2001, H. U. Güdel *et al.*^15^ confirmed that Cr^3+^, in association with Yb^3+^, creates VIS emission in the wavelength range from 400 to 700 nm.^16^ Moreover, our recent study^17^ showed that the Cr^3+^/Yb^3+^ co-doped in the glasses generates emission spectra in the wavelength regions of 420–700 nm, 660–860 nm, and 970–1150 nm corresponding to the excitations 358, 488, and 690 nm LD. Since then, we have been interested in enhancing emissions and optical properties of Cr^3+^/Yb^3+^ co-doped in the glasses.^17,18^ To this aim, embedding the coinage ions (such as, Ag^+^, Cu^+^ ions) into the glass through the ion exchange process^20–22^ is one of the different solutions which brought about positive results.^3,12,19^ In fact, the ion exchange process between coinage ions and alkali ions has many advantages compared with other traditional methods.^21^ For example, in many previous researches^22–24^ as well as in our recent work,^25^ it was shown that the coinage ions introduced by the ion exchange processes could significantly affect the optical properties of the glasses such as the refractive index, the optical density, the near-infrared emission spectrum, or even chemically strengthen the glasses^26,27^ as well as modify glass structure.^28^

Through the ion exchange process, the Cu^+^ and Cu^2+^ ions, as well as Ag^+^ ions, could be ejected into the surfaces of the glasses,^22,24,29–32^ then become neutral copper or/and silver atoms and grow into copper or/and silver nanoparticles (CuNPs or/and AgNPs).^24,29,32^ Therefore, the ion exchange process between copper or/and silver cations and alkali cations to enhance the luminescence of Cr^3+^/RE^3+^ co-doped has been studied in recent times.^21,29,33^ In this paper, we study the influences of the ion exchange process between copper and potassium on the optical bandgaps and spectroscopic properties of Cr^3+^/Yb^3+^ co-doped in 45SiO_2_–20Al_2_O_3_–12.5LaF_3_–10BaF_2_–9K_2_O–1Cr_2_O_3_–2.5Yb_2_O_3_ lanthanum aluminosilicate glasses. We calculated the values of both the direct and indirect optical bandgaps and figured out its manner of dependence on the salt concentration ratios between CuSO_4_:K_2_SO_4_. Besides, the energy transfer mechanism between Cu^+^ and Cu^2+^ ions with Cr^3+^, Yb^3+^ ions was also proposed and discussed.

## 2. Experimental details

In this study, we used the highly pure (99.99%) reagents of SiO_2_, Al_2_O_3_, LaF_3_, BaF_2_, Cr_2_O_3_, K_2_O, Yb_2_O_3_, K_2_SO_4_, and CuSO_4_ to prepare compositions of 45SiO_2_–20Al_2_O_3_–12.5LaF_3_–10BaF_2_–9K_2_O–1Cr_2_O_3_–2.5Yb_2_O_3_ (SALBK) by the conventional melting method. After being condensed in a platinum crucible, 12 g of the mixture of these materials were put into an electric furnace to be heated at 1600 °C for 1 h under air atmosphere. After that, we poured the molten mixture into a mold placed on a polished plate made from stainless steel to form glass samples, then annealed all of them at 450 °C for 6 h to remove thermal strains.^34^ We next cut them into 10 mm × 10 mm × 2 mm size and finally polished their surface for the sake of measurements.

In order to perform the ion exchange process between copper and potassium, we prepared salt mixtures with different concentration ratios between *x*CuSO_4_:(100 − *x*)K_2_SO_4_ where *x* varies as particularly given in Table 1.

**Table 1:** The concentration ratio of salts mixture for the ion exchange process

Name of glass samples	CuSO_4_*x* mol%	K_2_SO_4_(100 − *x*) mol%	Salt concentration ratio *p* between CuSO_4_ and K_2_SO_4_
SALBK-1Cr2.5Yb-0Cu	0	100	0
SALBK-1Cr2.5Yb-15Cu	15	85	0.18
SALBK-1Cr2.5Yb-20Cu	20	80	0.25
SALBK-1Cr2.5Yb-25Cu	25	75	0.33
SALBK-1Cr2.5Yb-30Cu	30	70	0.43
SALBK-1Cr2.5Yb-35Cu	35	65	0.54

All the experimental measurements with the glass samples, which were carried out at the ambient air temperature, and their corresponding instruments are listed in Table 2.

**Table 2:** The measurements and their corresponding instruments

Measurement	Instrument
Differential thermal analysis (DTA)	DTA-60AH Shimadzu
X-ray photoelectron spectroscopy (XPS) spectra	PHI5500 ESCA spectrometer
XRD analysis	Powder diffractometer (BRUKER AXS GMBH) using CuKα radiation
Absorption spectra	U-4100 Hitachi spectrophotometer
NIR emission spectra	SBP300 Zolix spectrophotometer with an InGaAs detector
Visible emission spectra	F-7000 Hitachi fluorescence spectrophotometer
Energy-dispersive X-ray spectroscopy (EDS)	Field emission scanning electron microscopy (FESEM)
Decay lifetimes	Edinburgh instruments FLS-1000 fluorescence spectrometer

**Fig. 1:**
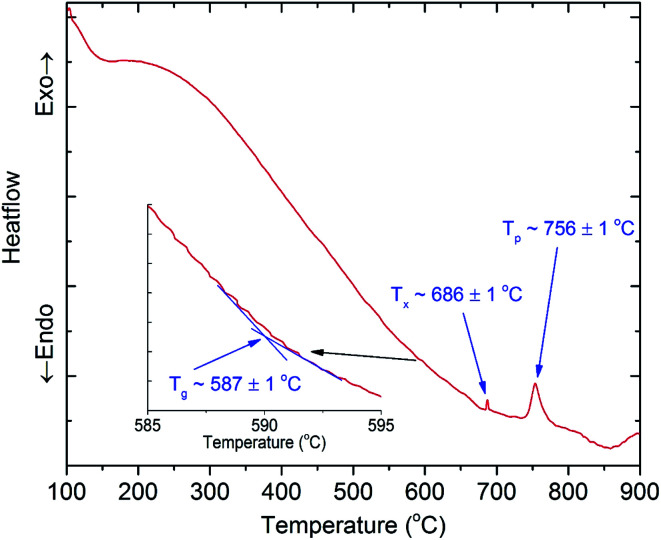
DTA analysis of 45SiO_2_–20Al_2_O_3_–12.5LaF_3_–10BaF_2_–9K_2_O–1Cr_2_O_3_–2.5Yb_2_O_3_ (SALBK) glass sample.

To find the suitable temperature ranges for the ion exchange process between copper and potassium, we conducted DTA analysis for 45SiO_2_–20Al_2_O_3_–12.5LaF_3_–10BaF_2_–9K_2_O–1Cr_2_O_3_–2.5Yb_2_O_3_ (SALBK) glass sample. The results are shown in Fig. 1, where *T*_g_, *T*_x_, and *T*_p_ are the glass transition temperature, the crystallization onset temperature and crystallization peak temperature, respectively. According to the results, we chose 587 °C as the temperature of the salt mixtures into which we submerged the glass samples for 24 h.^34^

Next, they were taken out and washed with alcohol and distilled water to eliminate all the residual salt on their surfaces. After all, the glass samples were further heat-treated at 686 °C for 8 h to promote the copper nanoparticles (CuNPs) formation.

## 3. Results and discussion

**Fig. 2:**
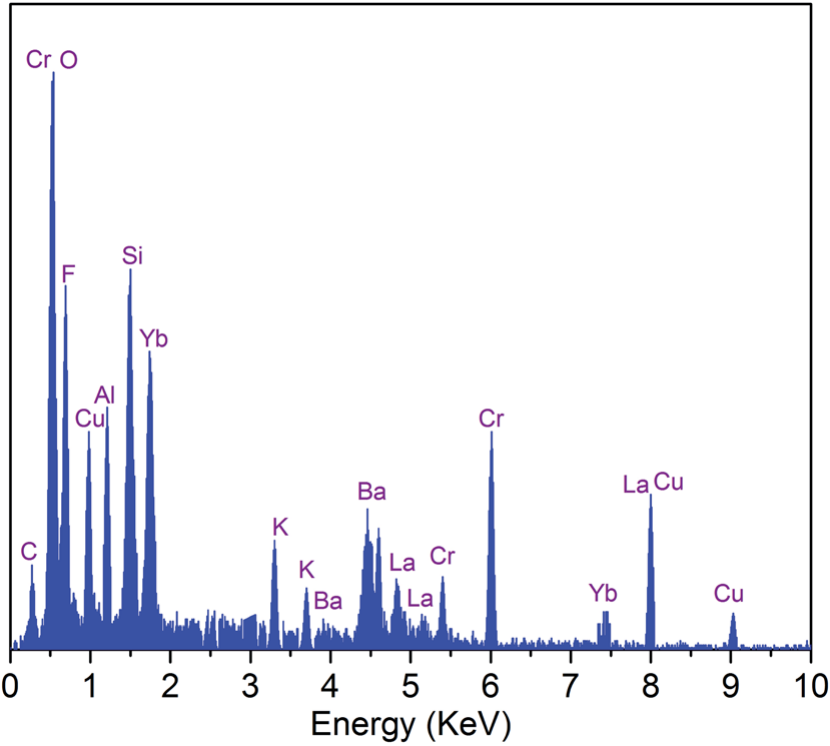
EDS analysis of SALBK-1Cr2.5Yb-35Cu glass sample.

The EDS analysis of the SALBK-1Cr2.5Yb-35Cu glass sample is shown in Fig. 2. In addition to the EDS peaks of the Si, O, Al, La, F, Ba, K, Cr, and Yb atoms in SALBK glass,^35^ the ones of copper were strongly obtained corresponding to the energy values about 0.95, 8.04, and 8.99 keV,^36^ which means that the copper and potassium ions have been added into the glass network through the ion exchange process.^37^

**Fig. 3:**
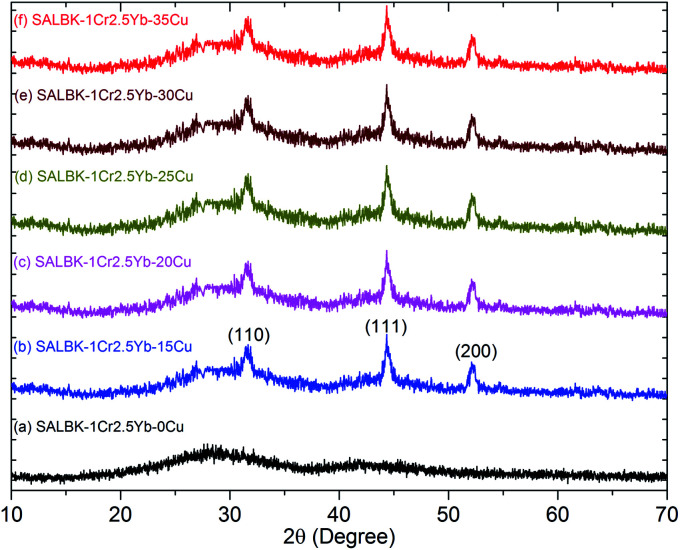
XRD analysis of SALBK-1Cr2.5Yb-0Cu, SALBK-1Cr2.5Yb-15Cu, SALBK-1Cr2.5Yb-20Cu, SALBK-1Cr2.5Yb-25Cu, SALBK-1Cr2.5Yb-30Cu, and SALBK-1Cr2.5Yb-35Cu glass samples.

To investigate the influence of the ion exchange process between copper and potassium on the structure of glass materials, we performed the XRD analysis of all the glass samples together with using X'Pert HighScore Plus software,^38^ showing the results in Fig. 3. Conspicuously, the XRD pattern of the SALBK-1Cr2.5Yb-0Cu glass sample has no diffraction peak,^39^ while all of the others show three main peaks at 2*θ* = 30.6 degree (110), 2*θ* = 44.2 degree (111) and 2*θ* = 52.1 degree (200)^40^ due to the formation of copper nanoparticles (CuNPs).^41^ Besides, no diffraction peak of other nanocrystals is observed for all the samples.

**Fig. 4:**
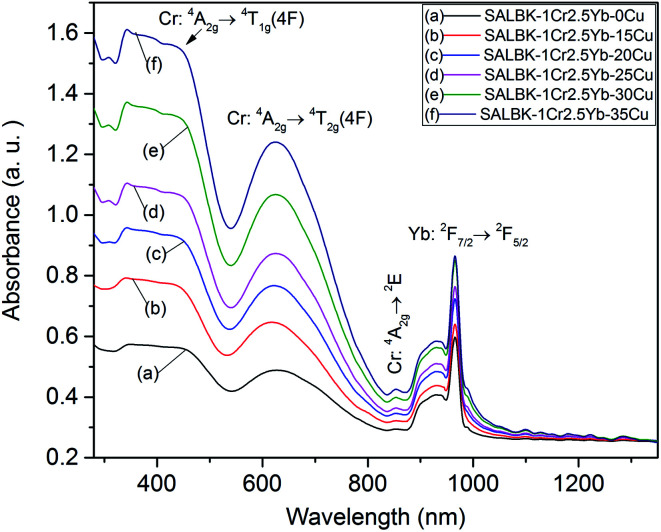
Absorption spectra of SALBK-1Cr2.5Yb-0Cu, SALBK-1Cr2.5Yb-15Cu, SALBK-1Cr2.5Yb-20Cu, SALBK-1Cr2.5Yb-25Cu, SALBK-1Cr2.5Yb-30Cu, and SALBK-1Cr2.5Yb-35Cu glass samples.

**Fig. 5:**
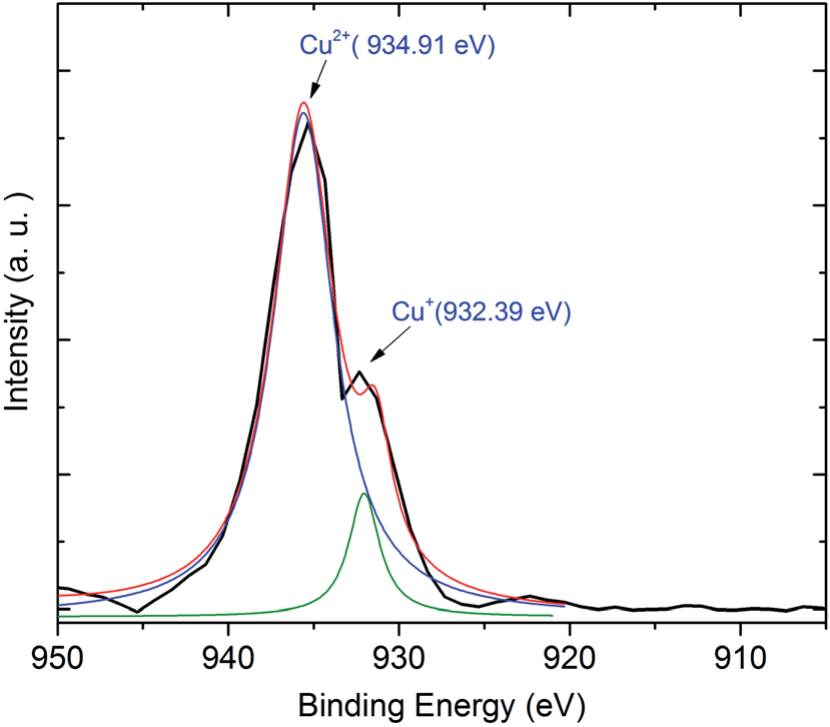
XPS spectra of SABLK-15Cu glass sample.

Next, we exhibit the absorption spectra of SALBK-1Cr2.5Yb-0Cu, SALBK-1Cr2.5Yb-15Cu, SALBK-1Cr2.5Yb-20Cu, SALBK-1Cr2.5Yb-25Cu, SALBK-1Cr2.5Yb-30Cu, and SALBK-1Cr2.5Yb-35Cu glass samples in Fig. 4. For the SALBK-1Cr2.5Yb-0Cu glass sample, we can observe three absorption peaks bands centered at 470, 650, and 878 nm, which is evidence that they are from the ^4^A_2g_ → ^4^T_1g_(F), ^4^A_2g_ → ^4^T_2g_(F) and ^4^A_2g_ → ^2^E transitions of Cr^3+^.^17,42,43^ In addition, with the increase in salt concentration ratio *p* from 0.18 to 0.54, the emission intensities of Cr^3+^/Yb^3+^ co-doped bands centered at 470, 650 nm, and 878 nm strongly increased. These increments may be ascribed to the local surface plasmon resonance (LSPR) of copper ions^44^ and the crystal field variation caused by copper ions.^17,43,45^ Moreover, the intensities of two absorption spectra bands centered at 306 nm and 878 nm also increased significantly, which confirms the existence and the role of Cu^+^ and Cu^2+^, respectively,^46^ in the absorption spectra of Cr^3+^/Yb^3+^ co-doped. The Cu^+^ formed and exists in silicate glass due to reducing of Cu^2+^.^47,48^ To further demonstrate the existence of both Cu^+^ and Cu^2+^ ions in the glass after ion exchange. We analyzed XPS spectra of the SABLK-15Cu glass sample, the results are shown in Fig. 5 (the blue and green curves are the curve-fitting Gaussian of XPS spectra at 934.91 eV and 932.39 eV, respectively). Based on these results, we can confirm the existence of both Cu^+^ and Cu^2+^ ions in the SALBK glasses, corresponding to the XPS major peaks at 932.39 eV and 934.91 eV.^49–52^

**Fig. 6:**
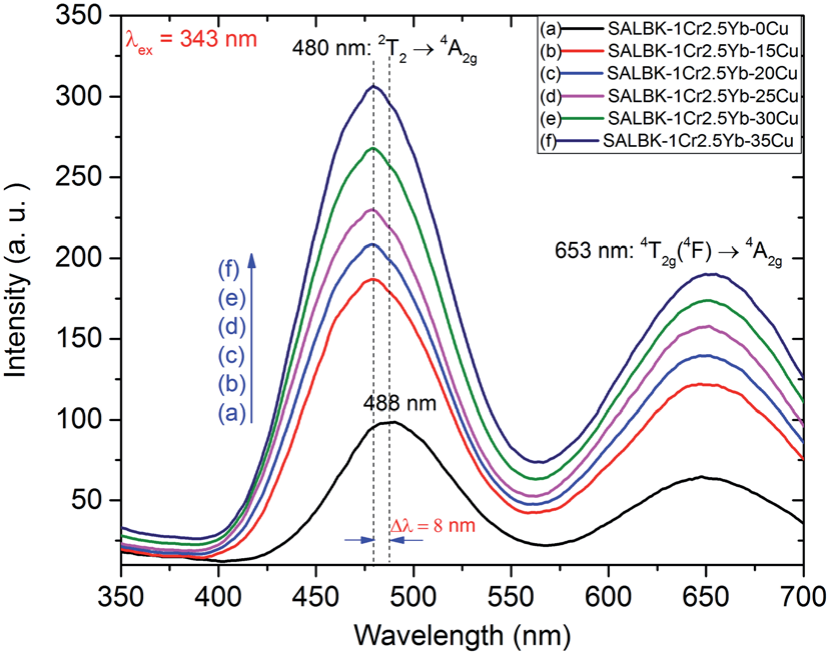
Visible emission spectra of SALBK-1Cr2.5Yb-0Cu, SALBK-1Cr2.5Yb-15Cu, SALBK-1Cr2.5Yb-20Cu, SALBK-1Cr2.5Yb-25Cu, SALBK-1Cr2.5Yb-30Cu, and SALBK-1Cr2.5Yb-35Cu glass samples under excitation 343 nm.

In Fig. 6, we present the visible emission spectra under excitation 343 nm of all these glass samples with two peaks at about 488 and 653 nm, which are assigned to the ^2^T_2_ → ^4^A_2g_ and ^4^T_2g_(^4^F) → ^4^A_2g_ transitions of Cr^3+^.^17,43,45^ As shown, the visible emission intensities of Cr^3+^/Yb^3+^ co-doped bands centered at 488 and 653 nm significantly increased with the increase in salt concentration ratio *p* from 0.18 to 0.54.^47^ Interestingly, the peak at 488 nm of Cr^3+^ under 343 nm excitation has a slight blue-shift of about 8 nm. The emission peak shift at 488 nm of Cr^3+^ is assigned to the role of Cu^+^ cations. This result is also consistent with the suggestions discussed in the paper^53^ of Tian-Shuai Lv *et al.*

The increases in visible emission intensity of Cr^3+^/Yb^3+^ co-doped band centered at 488 nm and 653 nm were assigned to the energy transfer (ET_1_) process from ^3^E_g_(T_2g_) → ^1^A_g_ transition of Cu^+^ to ^2^T_2_ → ^4^A_2g_ transition of Cr^3+^,^47,48^ and to the ET_2_ process from ^3^E_g_(T_1g_) → ^1^A_g_ transition of Cu^+^ to ^4^T_2g_(^4^F) → ^4^A_2g_ transition of Cr^3+^,^47,48^ respectively. These two ET_1_, ET_2_ processes are proposed as follows:

ET_1_: ^3^E_g_(T_2g_)(Cu^+^) + ^4^A_2g_(Cr^3+^) → ^2^T_2_(Cr^3+^) + ^1^A_g_(Cu^+^).

ET_2_: ^3^E_g_(T_1g_)(Cu^+^) + ^4^A_2g_(Cr^3+^) → ^4^T_2g_(^4^F)(Cr^3+^) + ^1^A_g_(Cu^+^).

**Fig. 7:**
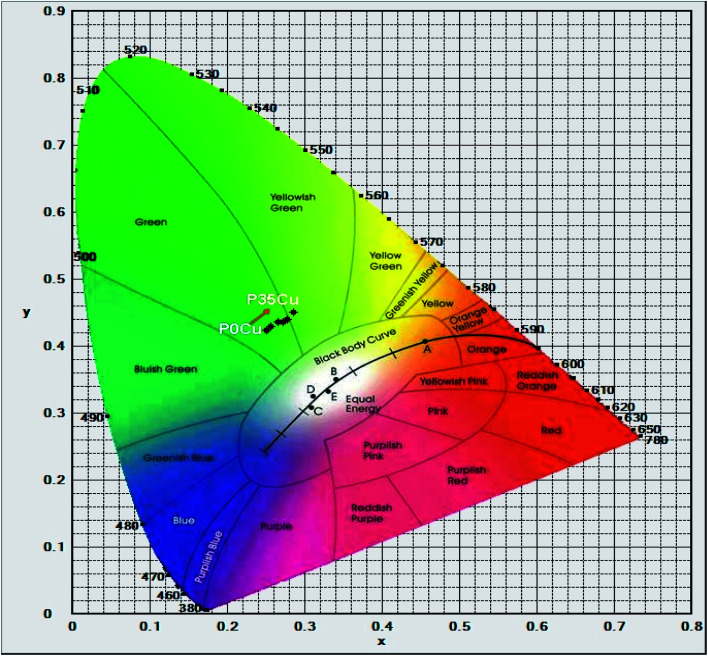
CIE 1931(*x*; *y*) chromaticity coordinates for luminescence of Cr^3+^/Yb^3+^ co-doped in SALBK-1Cr2.5Yb-0Cu, SALBK-1Cr2.5Yb-15Cu, SALBK-1Cr2.5Yb-20Cu, SALBK-1Cr2.5Yb-25Cu, SALBK-1Cr2.5Yb-30Cu, and SALBK-1Cr2.5Yb-35Cu glass samples.

Furthermore, the calculated results of the CIE 1931(*x*; *y*) chromaticity coordinates for luminescence of Cr^3+^/Yb^3+^ co-doped in SALBK-1Cr2.5Yb-0Cu, SALBK-1Cr2.5Yb-15Cu, SALBK-1Cr2.5Yb-20Cu, SALBK-1Cr2.5Yb-25Cu, SALBK-1Cr2.5Yb-30Cu, and SALBK-1Cr2.5Yb-35Cu glass samples in correspondence to the P0Cu, P15Cu, P20Cu, P25Cu, P30Cu, and P35Cu points on the CIE 1931(*x*, *y*) chromaticity coordinates are given in Table 3, while Fig. 7 is for the sake of illustration. It can be seen that except that the CIE 1931(*x*; *y*) chromaticity coordinates for luminescence of Cr^3+^/Yb^3+^ co-doped in SALBK-1Cr2.5Yb-35Cu glass sample were shifted to the yellowish-green color region, the remaining were originally in the green color region.

**Table 3:** CIE 1931 (*x*; *y*) chromaticity coordinates for luminescence of Cr^3+^/Yb^3+^ co-doped in SALBK-1Cr2.5Yb-0Cu, SALBK-1Cr2.5Yb-15Cu, SALBK-1Cr2.5Yb-20Cu, SALBK-1Cr2.5Yb-25Cu, SALBK-1Cr2.5Yb-30Cu, and SALBK-1Cr2.5Yb-35Cu glass samples

Name of glass samples	CIE*x*	CIE*y*	Position on the CIE 1931(*x*, *y*) chromaticity coordinates	Color region
SALBK-1Cr2.5Yb-0Cu	0.2511	0.4241	P0Cu	Green
SALBK-1Cr2.5Yb-15Cu	0.2648	0.4356	P15Cu	Green
SALBK-1Cr2.5Yb-20Cu	0.2562	0.4294	P20Cu	Green
SALBK-1Cr2.5Yb-25Cu	0.2722	0.4363	P25Cu	Green
SALBK-1Cr2.5Yb-30Cu	0.2779	0.4402	P30Cu	Green
SALBK-1Cr2.5Yb-35Cu	0.2851	0.4501	P35Cu	Yellowish-green

**Fig. 8:**
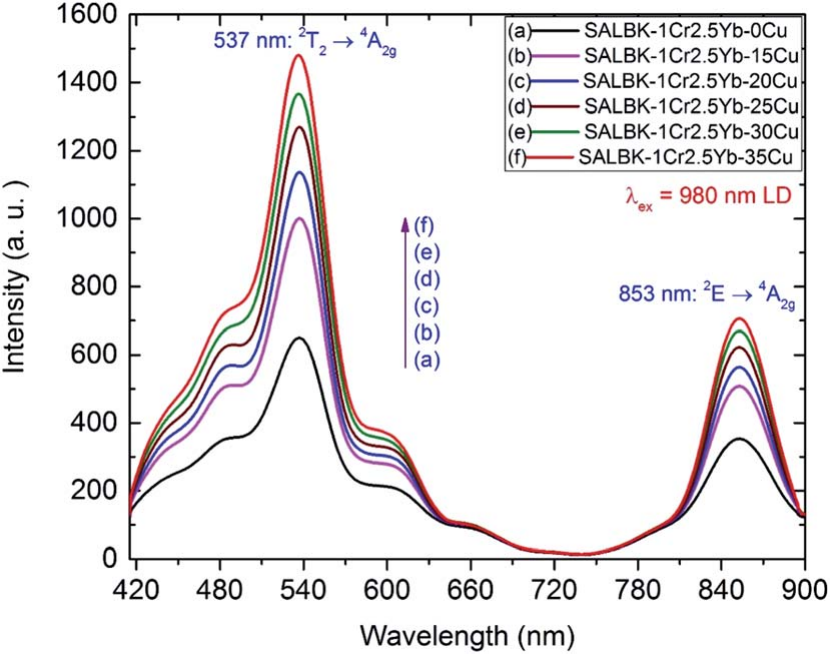
UC spectra of Cr^3+^/Yb^3+^ co-doped in SALBK-1Cr2.5Yb-0Cu, SALBK-1Cr2.5Yb-15Cu, SALBK-1Cr2.5Yb-20Cu, SALBK-1Cr2.5Yb-25Cu, SALBK-1Cr2.5Yb-30Cu, and SALBK-1Cr2.5Yb-35Cu glass samples under excitation 980 nm LD.

Fig. 8 shows the upconversion (UC) emission spectra of Cr^3+^/Yb^3+^ co-doped in all the glass samples under excitation 980 nm LD with two bands centered at ∼537 and 853 nm corresponding to ^2^T_2_ → ^4^A_2g_, and ^2^E → ^4^A_2g_ transitions of Cr^3+^, respectively.^17,45,54–56^ With the increase in salt concentration ratio *p* from 0.18 to 0.54, the emission intensities of these bands markedly increased. These increments were assigned to the aforementioned ET_1_ process (for the band centered at 537 nm) and to the ET_3_ process from ^2^B_2g_ → ^2^B_1g_ transition of Cu^2+^ to ^2^E → ^4^A_2g_ transition of Cr^3+^ (for the one at 853 nm),^5,46^ which is proposed as follows:

ET_3_: ^2^E_g_(Cu^2+^) + ^4^A_2g_(Cr^3+^) → ^2^E(Cr^3+^) + ^2^B_1g_(Cu^2+^).

**Fig. 9:**
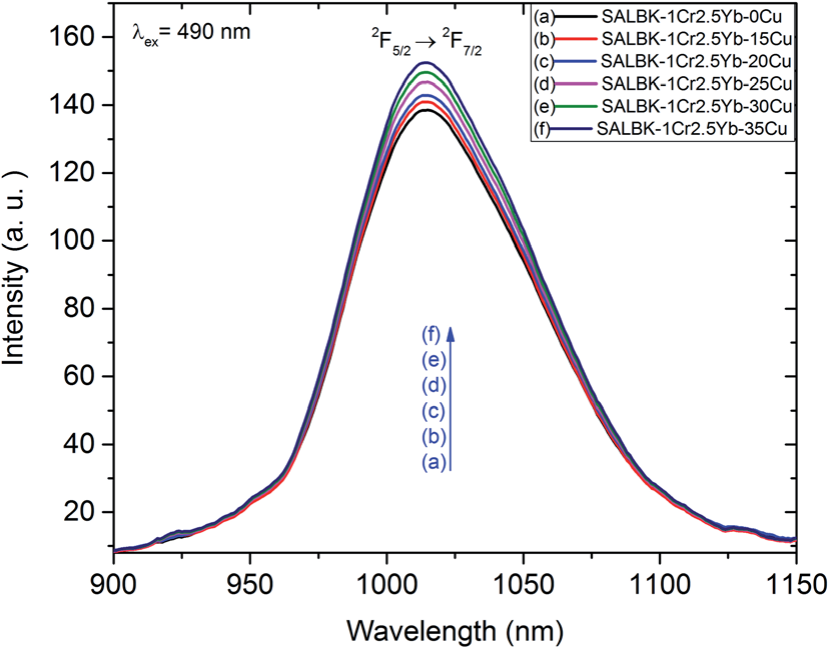
NIR emission spectra of Cr^3+^/Yb^3+^ co-doped in SALBK-1Cr2.5Yb-0Cu, SALBK-1Cr2.5Yb-15Cu, SALBK-1Cr2.5Yb-20Cu, SALBK-1Cr2.5Yb-25Cu, SALBK-1Cr2.5Yb-30Cu, and SALBK-1Cr2.5Yb-35Cu glass samples, excited by 490 nm.

Likewise, Fig. 9 shows the NIR emission spectra under excitation 490 nm. There was only one NIR emission peak at 1016 nm, and it is assigned to the ^2^F_5/2_ → ^2^F_7/2_ transition of Yb^3+^.^3,5^ Similarly, the NIR emission intensity of the band centered at 1016 nm also increased with the increase in salt concentration ratio p, which is a manifestation of the ET_4_ process from ^2^B_2g_ → ^2^B_1g_ transition of Cu^2+^ to ^2^F_5/2_ → ^2^F_7/2_ transition of Yb^3+^:^57^

ET_4_: ^2^E_g_(Cu^2+^) + ^2^F_7/2_(Yb^3+^) → ^2^F_5/2_(Yb^3+^) + ^2^B_1g_(Cu^2+^).

**Fig. 10:**
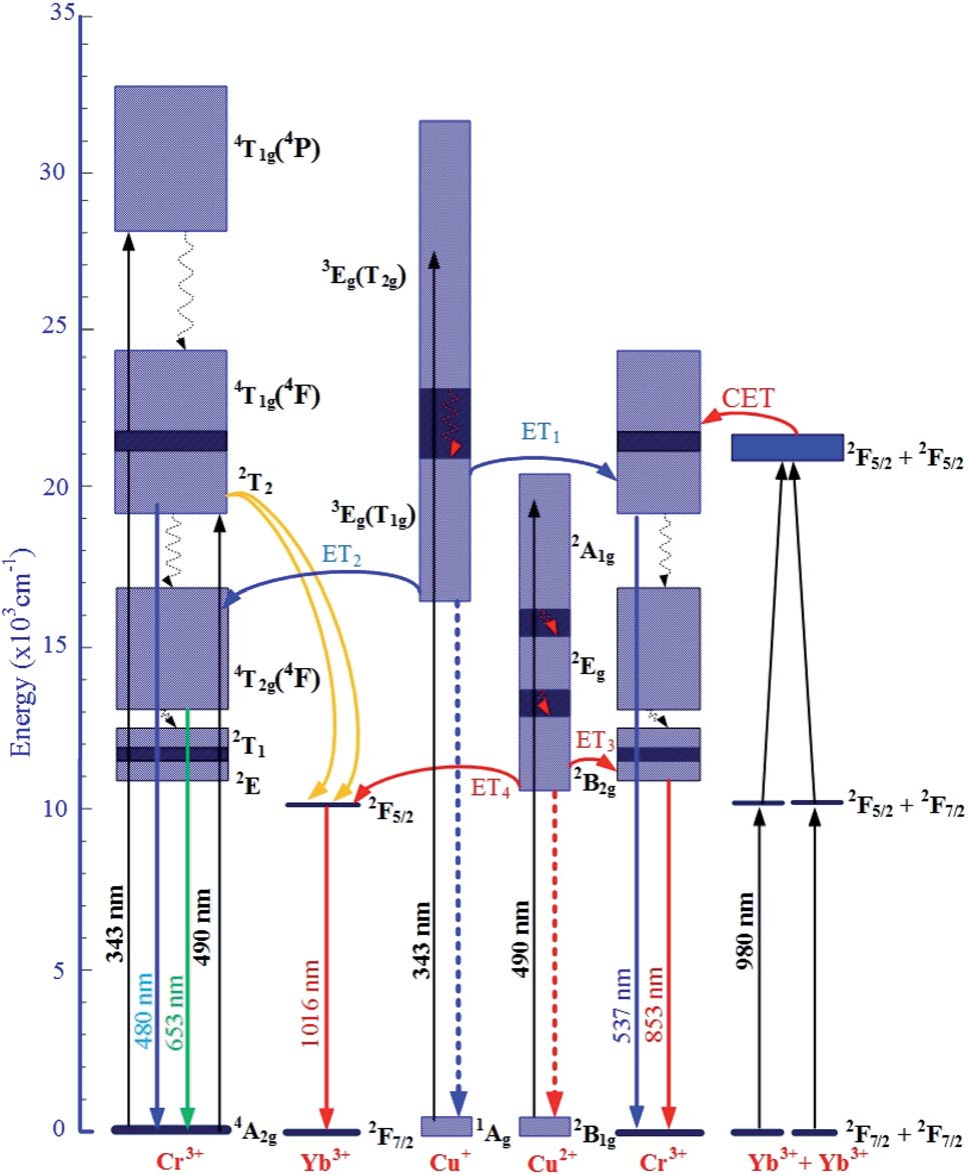
Mechanism ET processes for the visible, UC, and NIR luminescence of Cr^3+^/Yb^3+^ co-doped under excitations 343, 490, and 980 nm LD.

The mechanism for the visible and NIR luminescence of Cr^3+^/Yb^3+^ co-doped under excitations of 343, 490, and 980 nm LD, and the details of all the above-mentioned ET_1_, ET_2_, ET_3_, and ET_4_ processes are described in Fig. 10.

**Fig. 11:**
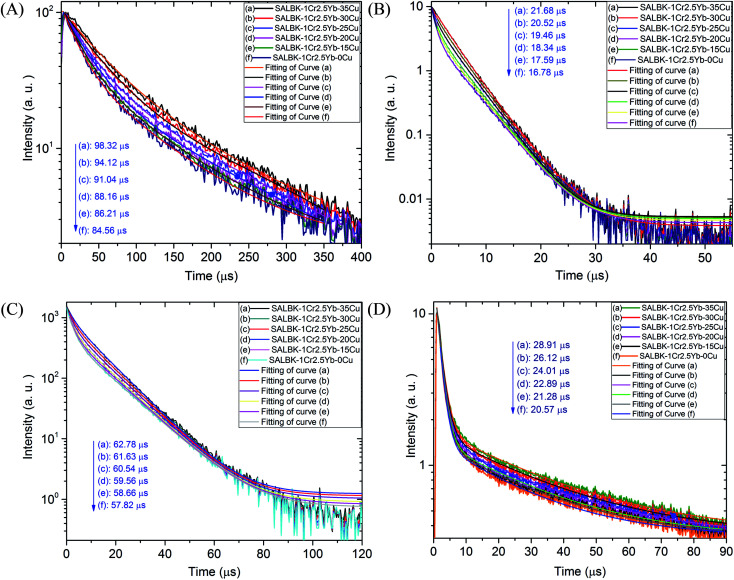
(A) Decay lifetimes curves of Cr^3+^ at 537 nm in SALBK-0Cu, SALBK-15Cu, SALBK-20Cu, SALBK-25Cu, SALBK-30Cu, and SALBK-35Cu glass samples, under excitation 980 nm. (B) Decay lifetimes curves of Cr^3+^ at 653 nm in SALBK-0Cu, SALBK-15Cu, SALBK-20Cu, SALBK-25Cu, SALBK-30Cu, and SALBK-35Cu glass samples, under excitation 343 nm. (C) Decay lifetimes curves of Cr^3+^ at 853 nm in SALBK-0Cu, SALBK-15Cu, SALBK-20Cu, SALBK-25Cu, SALBK-30Cu, and SALBK-35Cu glass samples, under excitation 980 nm LD. (D) Decay lifetimes curves of Yb^3+^ at 1016 nm in SALBK-0Cu, SALBK-15Cu, SALBK-20Cu, SALBK-25Cu, SALBK-30Cu, and SALBK-35Cu glass samples, under excitation 490 nm.

To further validate evidence for the ET_1_, ET_2_, ET_3_, ET_4_ processes from Cu^+^/Cu^2+^ ions to Cr^3+^ and Yb^3+^ ions, we carried out the decay lifetimes measurement for several glass samples. Namely, the decay lifetimes curves of Cr^3+^ at 537, 653, and 853 nm to demonstrations for the ET_1_, ET_2_, ET_3_ processes are shown in Fig. 11A–C, respectively while the decay lifetimes curve of Yb^3+^ at 1016 nm to demonstrations for the ET_4_ is in Fig. 11D.

We recall that the decay lifetimes can be calculated by the following equation:^58,59^1
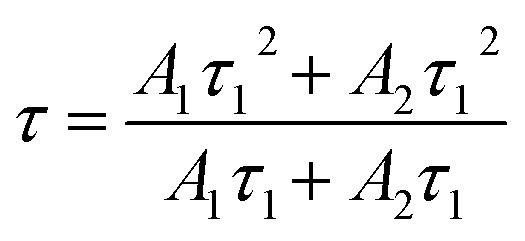
where *A*_1_, *A*_2_ are constants; *τ*_1_, *τ*_2_ are rapid and slow lifetimes for exponential components, respectively. Using the formula (1) for the data obtained in Fig. 11A–D, we calculated the decay lifetime values of Cr^3+^ and Yb^3+^, corresponding to the ET_1_, ET_2_, ET_3_ and ET_4_ processes, details are presented in Table 4.

**Table 4:** Decay lifetimes values of Cr^3+^ and Yb^3+^ demonstrating for the ET_1_, ET_2_, ET_3_, and ET_4_ processes

Name of glass samples	Decay lifetimes of Cr^3+^ at 537 nm, *λ*_ex_ = 980 nm LD (ET_1_ process)	Decay lifetimes of Cr^3+^ at 653 nm, *λ*_ex_ = 343 nm (ET_2_ process)	Decay lifetimes of Cr^3+^ at 853 nm, *λ*_ex_ = 980 nm LD (ET_3_ process)	Decay lifetimes of Yb^3+^ at 1016 nm, *λ*_ex_ = 490 nm (ET_4_ process)
SALBK-1Cr2.5Yb-35Cu	98.32 μs	21.68 μs	62.78 μs	28.91 μs
SALBK-1Cr2.5Yb-30Cu	94.12 μs	20.52 μs	61.63 μs	26.12 μs
SALBK-1Cr2.5Yb-25Cu	91.04 μs	19.46 μs	60.54 μs	24.01 μs
SALBK-1Cr2.5Yb-20Cu	88.16 μs	18.34 μs	59.56 μs	22.89 μs
SALBK-1Cr2.5Yb-15Cu	86.21 μs	17.59 μs	58.66 μs	21.28 μs
SALBK-1Cr2.5Yb-0Cu	84.56 μs	16.78 μs	57.82 μs	20.57 μs

Finally, we calculated and analyzed the optical bandgap (*E*_g_) of Cr^3+^/Yb^3+^ co-doped in SALBK glasses to evaluate whether and how it is affected by the ion exchange process between copper and potassium. The optical bandgap *E*_g_ can be calculated using Tauc's formula:^60^

**Fig. 12:**
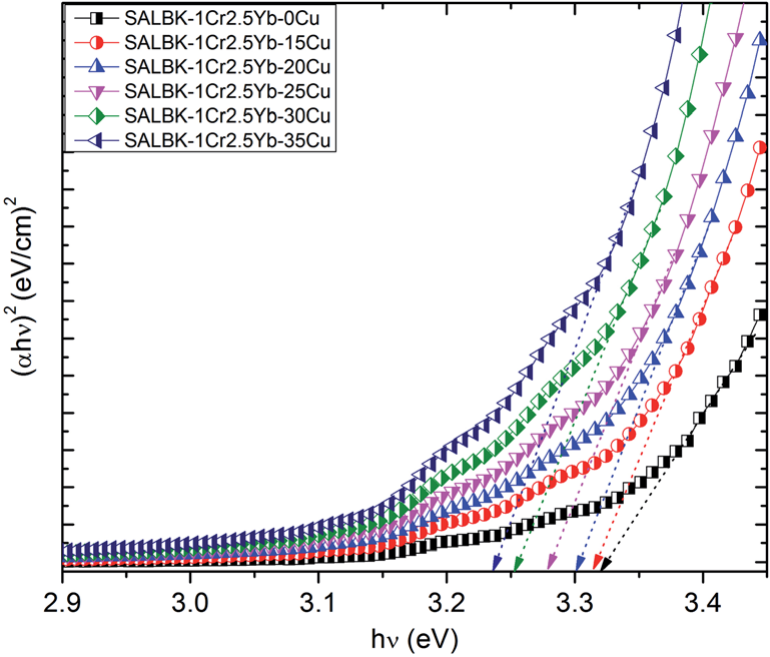
Plot of (*hν*) *versus* (*αhν*)^2^ for estimating the *E*_g_ of SALBK-1Cr2.5Yb-0Cu, SALBK-1Cr2.5Yb-15Cu, SALBK-1Cr2.5Yb-20Cu, SALBK-1Cr2.5Yb-25Cu, SALBK-1Cr2.5Yb-30Cu, and SALBK-1Cr2.5Yb-35Cu glass samples.

**Fig. 13:**
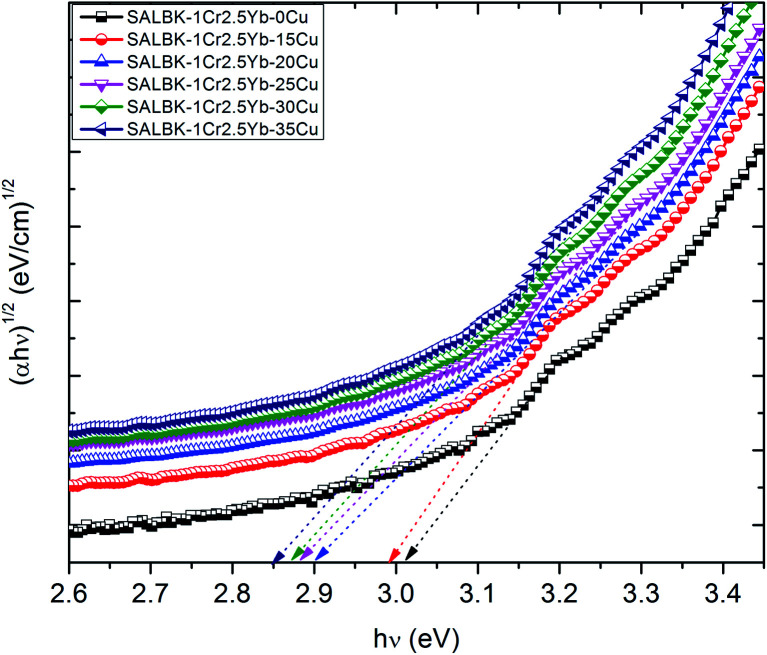
Plot of (*hν*) *versus* (*αhν*)^1/2^ for estimating the *E*_g_ of SALBK-1Cr2.5Yb-0Cu, SALBK-1Cr2.5Yb-15Cu, SALBK-1Cr2.5Yb-20Cu, SALBK-1Cr2.5Yb-25Cu, SALBK-1Cr2.5Yb-30Cu, and SALBK-1Cr2.5Yb-35Cu glass samples.

2 *αhν* = *A*(*hν* − *E*_g_)^*m*^

where *α* is the absorption coefficient, *A* is a constant, *m* is the power depending on the nature of transition (*m* = 1/2 for direct transition, and *m* = 2 for indirect transition).^61–63^ From formula (2), the values of both types (direct and indirect) of the optical bandgap in SALBK glasses are calculated and detailed in Table 5. For the sake of demonstration, we also present the plot of (*hν*) *versus* both (*αhν*)^2^ and (*αhν*)^1/2^ for estimating *E*_g_ of all the considered samples in Fig. 12 and 13.

**Table 5:** The values of both the direct and indirect optical bandgaps in SALBK glasses

Glass samples	Direct bandgap *hν* [(eV)]	Indirect bandgap *hν* [(eV)]
SALBK-1Cr2.5Yb-0Cu	3.33	3.02
SALBK-1Cr2.5Yb-15Cu	3.32	2.98
SALBK-1Cr2.5Yb-20Cu	3.30	2.91
SALBK-1Cr2.5Yb-25Cu	3.28	2.88
SALBK-1Cr2.5Yb-30Cu	3.25	2.87
SALBK-1Cr2.5Yb-35Cu	3.23	2.83

The calculation gave us the estimated results of 3.23–3.33 eV for direct bandgap and 2.83–3.02 eV for indirect bandgap. On the other hand, both bandgap types decrease when the salt concentration ratio *p* increases from 0.18 to 0.54. Thus, we can conclude that the ion exchange process between copper and potassium had a diminishing effect on the bandgap. The reasons for this effect can be: (i) Because there are non-bridging oxygens (NBOs) in the silicate glasses network,^64^ the Al^3+^ ions have some options to link with SiO_4_ tetrahedra to form (Al, Si)–O–Si, (Al, Si)–O–Al bonds,^65^ or with others groups to form (Al, Si)–O bonds.^65^ After the copper ions were introduced into the silica network by the copper-potassium ion exchange process, these bonds may be broken and then Al–O^−^ and Si–O^−^ groups can combine with copper ions to create the Si–O–Cu, and Al–O–Cu bonds. (ii) With the increase of salt concentration ratio, more negative sites appeared due to the local structure of the NBOs in the silicate glasses network.^64,66^

## 4. Conclusions

The CuNPs were formed in 45SiO_2_–20Al_2_O_3_–12.5LaF_3_–10BaF_2_–9K_2_O–1Cr_2_O_3_–2.5Yb_2_O_3_ lanthanum aluminosilicate glasses through the ion exchange process between copper and potassium process. The intensities of all three visible, UC, and NIR emissions of Cr^3+^/Yb^3+^ co-doped bands at 480, 537, 653, 853, and 1016 nm increased with the increase in the ratio of salt concentrations CuSO_4_:K_2_SO_4_ from 0.18 to 0.54. When the salt concentration ratio of the ion exchange process achieved 35 mol%CuSO_4_:65 mol%K_2_SO_4_, the CIE 1931(*x*; *y*) chromaticity coordinates for the luminescence of Cr^3+^/Yb^3+^ co-doped in SALBK-1Cr2.5Yb-35Cu glass sample shifted to the yellowish-green color region. At the same time, the estimated results of the optical bandgap (*E*_g_) confirmed that with the increase in the ratio of salt concentrations CuSO_4_:K_2_SO_4_ from 0.18 to 0.54, the value of both direct and indirect bandgaps decrease. Besides, the possible energy transfer processes from Cu^+^, Cu^2+^ ions to Cr^3+^, Yb^3+^ ions were determined through the experimental results of the spectroscopic emissions and the decay lifetimes.

## Conflicts of interest

There are no conflicts to declare.

## Supplementary Material

RA-011-D0RA10831F-s001
