# Supplementary material for: Influences of copper–potassium ion exchange process on the optical bandgaps and spectroscopic properties of Cr3+/Yb3+ co-doped in lanthanum aluminosilicate glasses
Source: RSC Adv. 2021 Feb 26;11(15):8917–26. doi: 10.1039/d0ra10831f (PMC8695246; doi:10.1039/d0ra10831f)
Supplement: RA-011-D0RA10831F-s001 [file RA-011-D0RA10831F-s001.pdf]

Electronic Supplementary Material (ESI) for RSC Advances.
This journal is © The Royal Society of Chemistry 2021

Electronic Supplementary Information

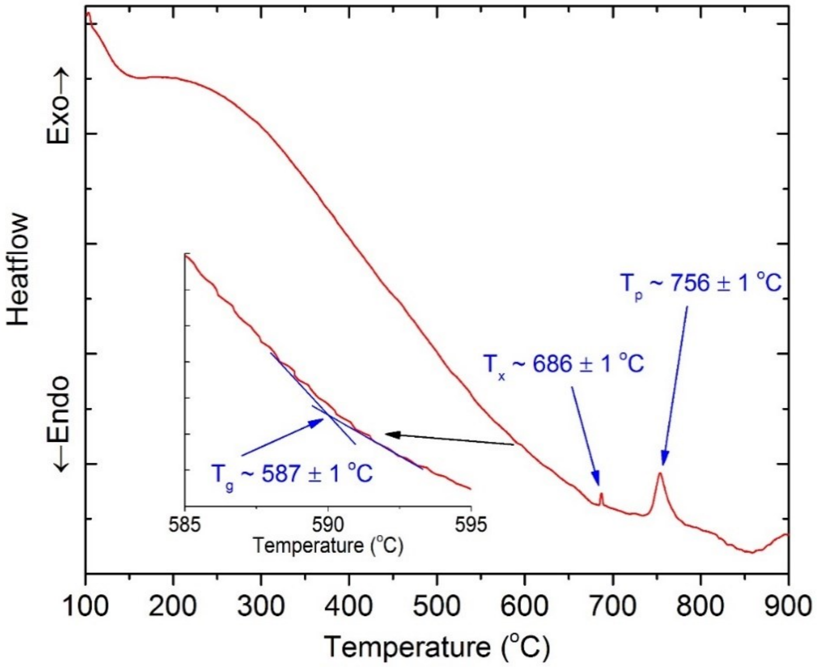

Fig. S1 DTA analysis of $45SiO_2–20Al_2O_3–12.5LaF_3–10BaF_2–9K_2O–1Cr_2O_3–2.5Yb_2O_3$ (SALBK) glass sample.

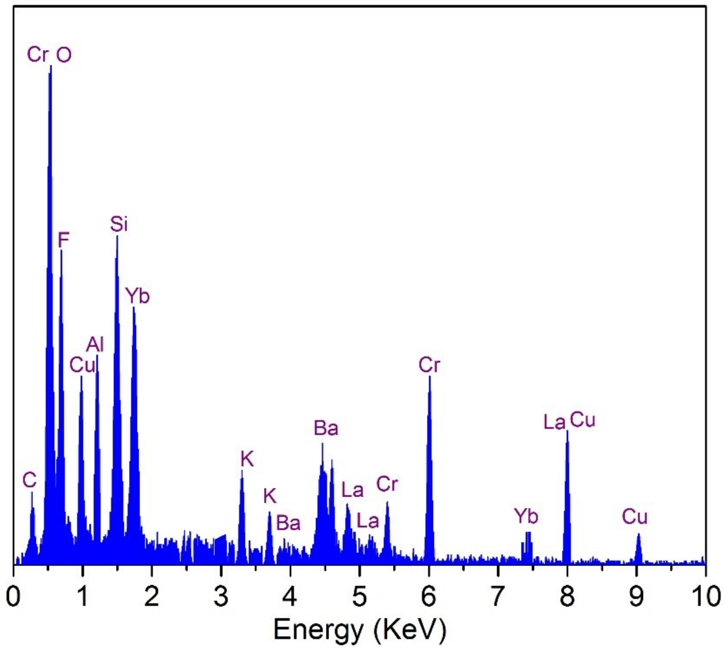

Fig. S2 EDS analysis of SALBK-1Cr2.5Yb-35Cu glass sample.

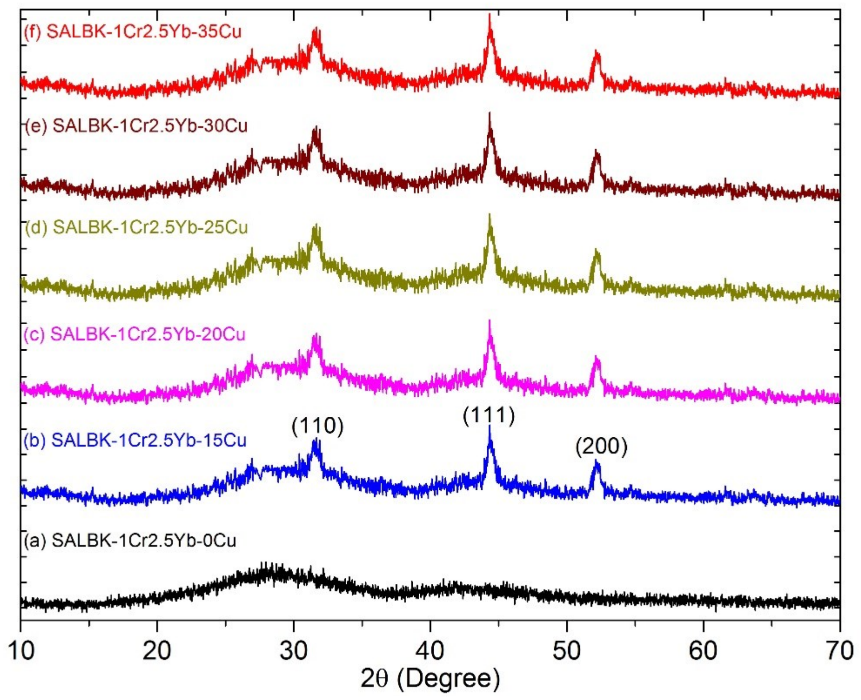

Fig. S3 XRD analysis of SALBK-1Cr2.5Yb-0Cu, SALBK-1Cr2.5Yb-15Cu, SALBK-1Cr2.5Yb-20Cu, SALBK-1Cr2.5Yb-25Cu, SALBK-1Cr2.5Yb-30Cu, and SALBK-1Cr2.5Yb-35Cu glass samples.

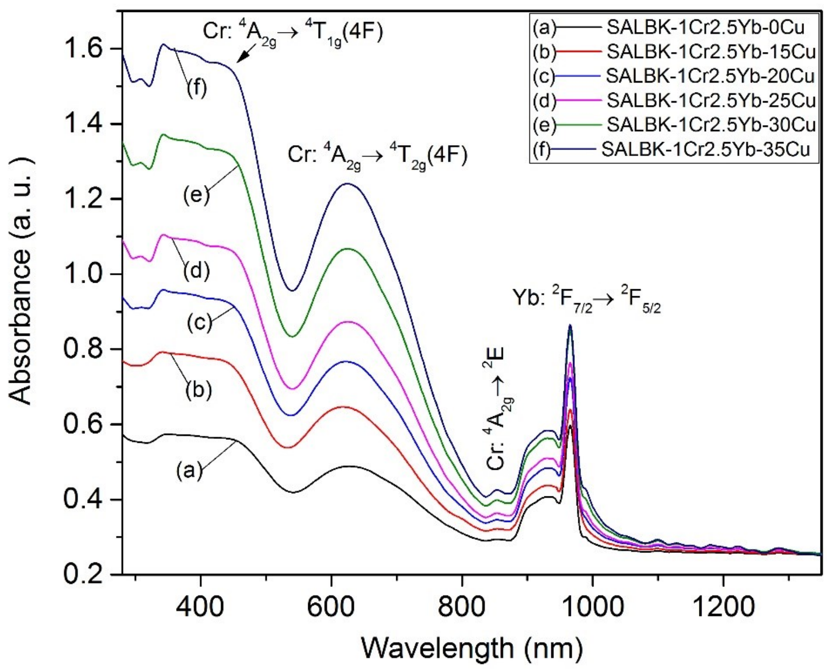

Fig. S4 Absorption spectra of SALBK-1Cr2.5Yb-0Cu, SALBK-1Cr2.5Yb-15Cu, SALBK-1Cr2.5Yb-20Cu, SALBK-1Cr2.5Yb-25Cu, SALBK-1Cr2.5Yb-30Cu, and SALBK-1Cr2.5Yb-35Cu glass samples.

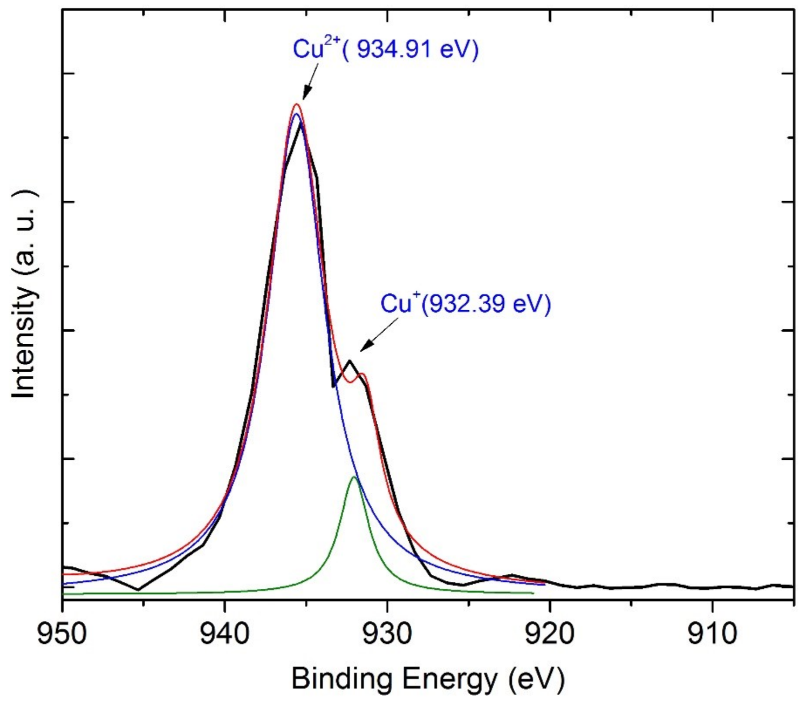

Fig. S5 XPS spectra of SABLK-15Cu glass sample.

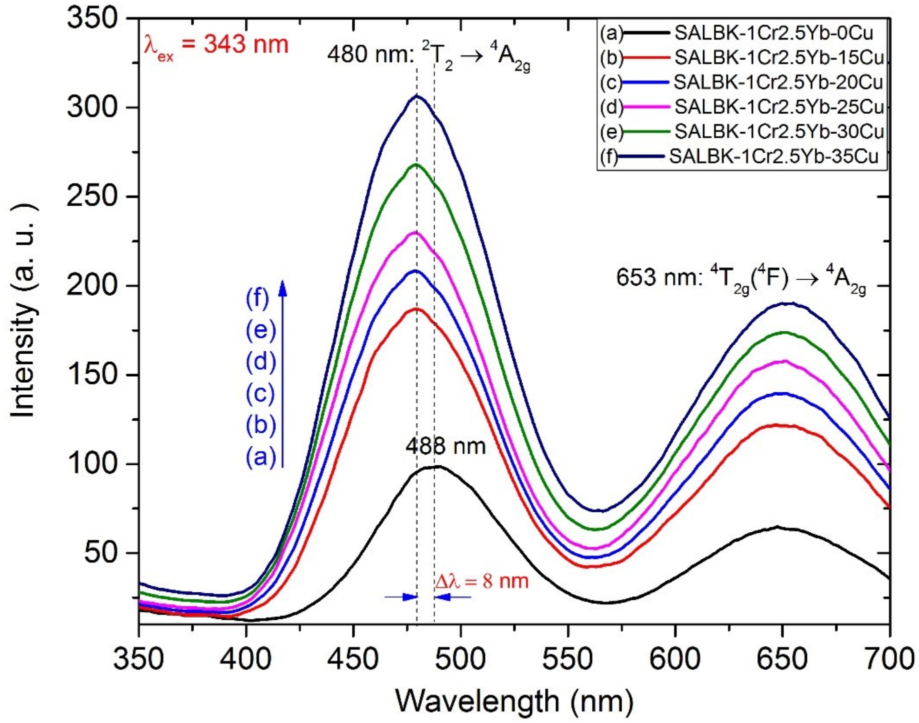

Fig. S6 Visible emission spectra of SALBK-1Cr2.5Yb-0Cu, SALBK-1Cr2.5Yb-15Cu, SALBK-1Cr2.5Yb-20Cu, SALBK-1Cr2.5Yb-25Cu, SALBK-1Cr2.5Yb-30Cu, and SALBK-1Cr2.5Yb-35Cu glass samples under excitation 343 nm.

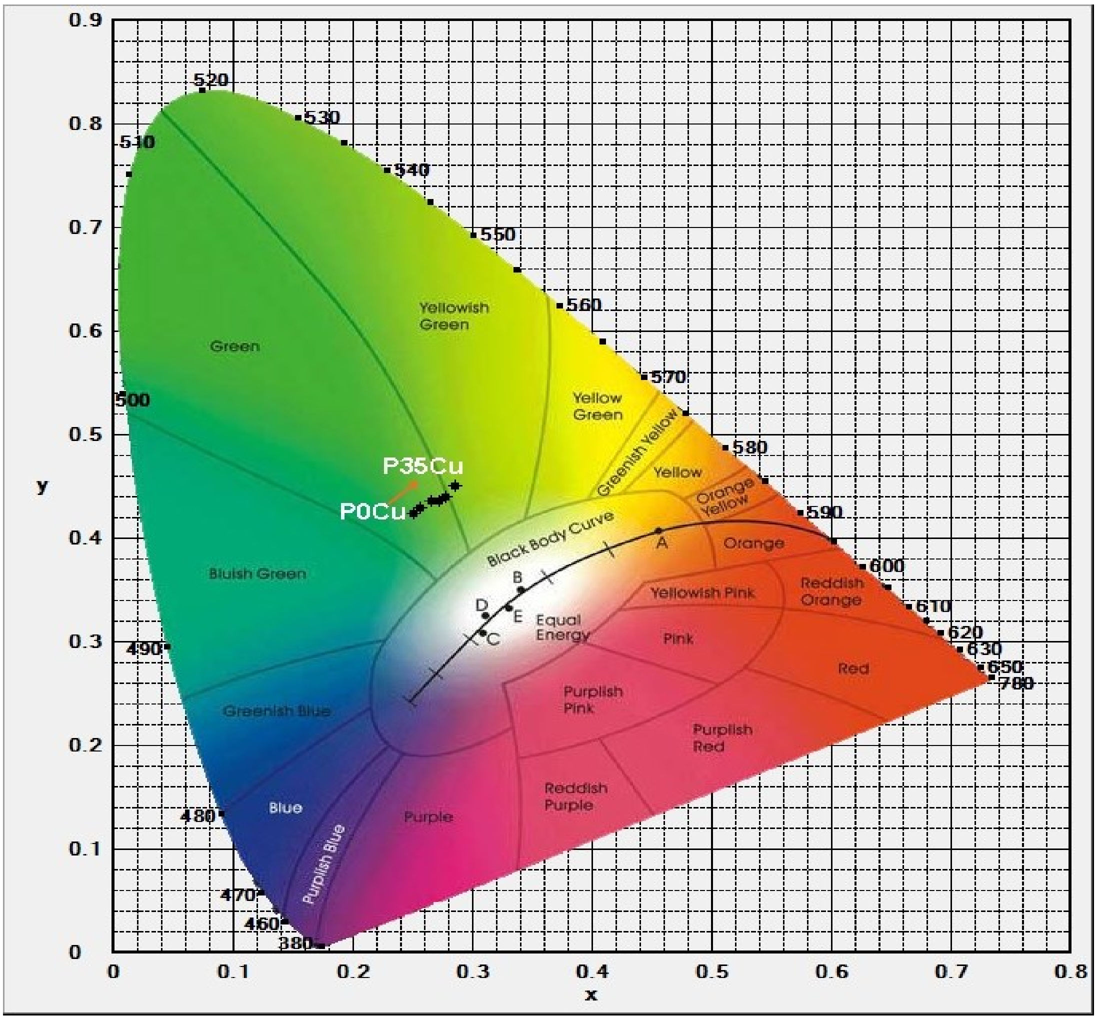

Fig. S7 CIE 1931 (x; y) chromaticity coordinates for luminescence of Cr^{3+}/Yb^{3+} co-doped in SALBK-1Cr2.5Yb-0Cu, SALBK-1Cr2.5Yb-15Cu, SALBK-1Cr2.5Yb-20Cu, SALBK-1Cr2.5Yb-25Cu, SALBK-1Cr2.5Yb-30Cu, and SALBK-1Cr2.5Yb-35Cu glass samples.

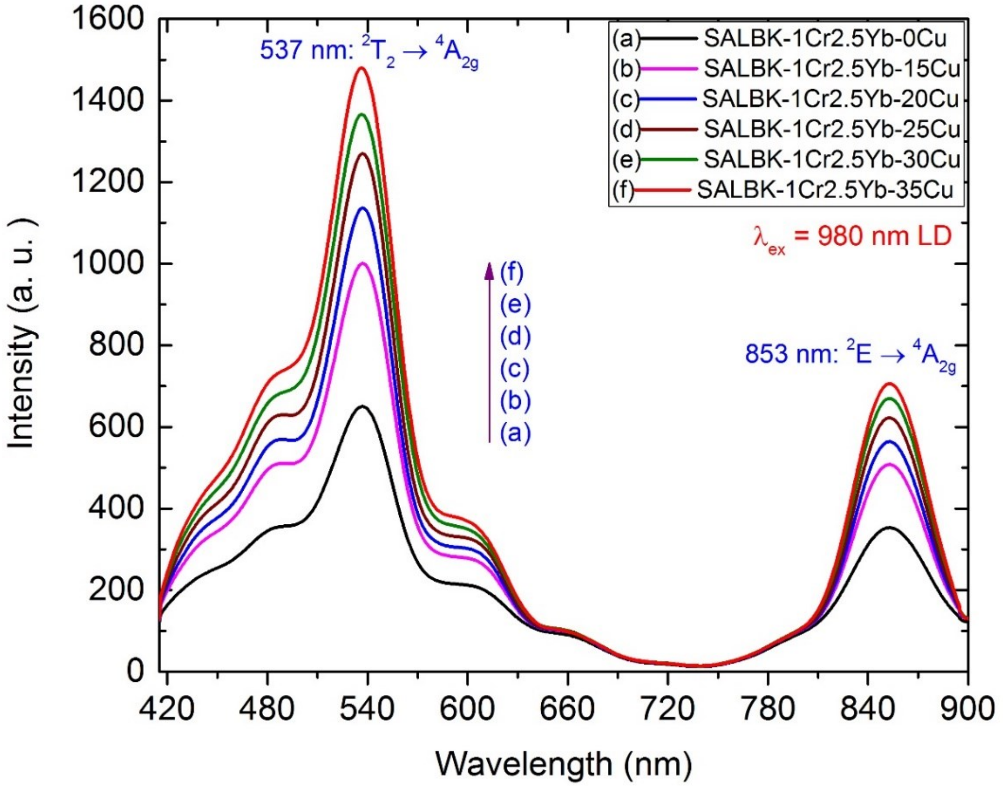

Fig. S8 UC spectra of Cr^{3+}/Yb^{3+} co-doped in SALBK-1Cr2.5Yb-0Cu, SALBK-1Cr2.5Yb-15Cu, SALBK-1Cr2.5Yb-20Cu, SALBK-1Cr2.5Yb-25Cu, SALBK-1Cr2.5Yb-30Cu, and SALBK-1Cr2.5Yb-35Cu glass samples under excitation 980 nm LD.

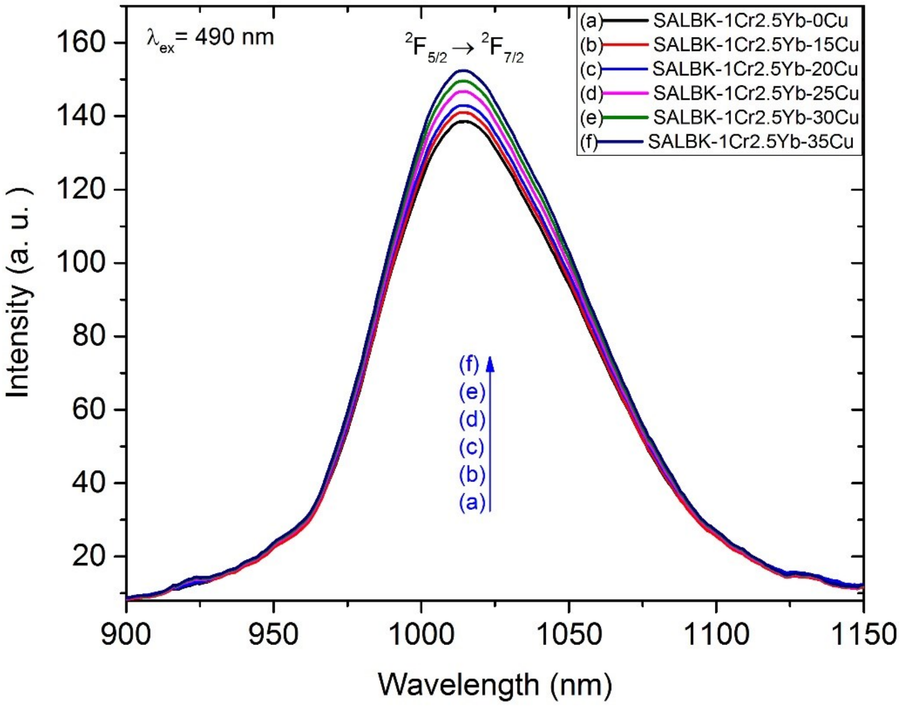

Fig. S9 NIR emission spectra of Cr^{3+}/Yb^{3+} co-doped in SALBK-1Cr2.5Yb-0Cu, SALBK-1Cr2.5Yb-15Cu, SALBK-1Cr2.5Yb-20Cu, SALBK-1Cr2.5Yb-25Cu, SALBK-1Cr2.5Yb-30Cu, and SALBK-1Cr2.5Yb-35Cu glass samples, excited by 490 nm.

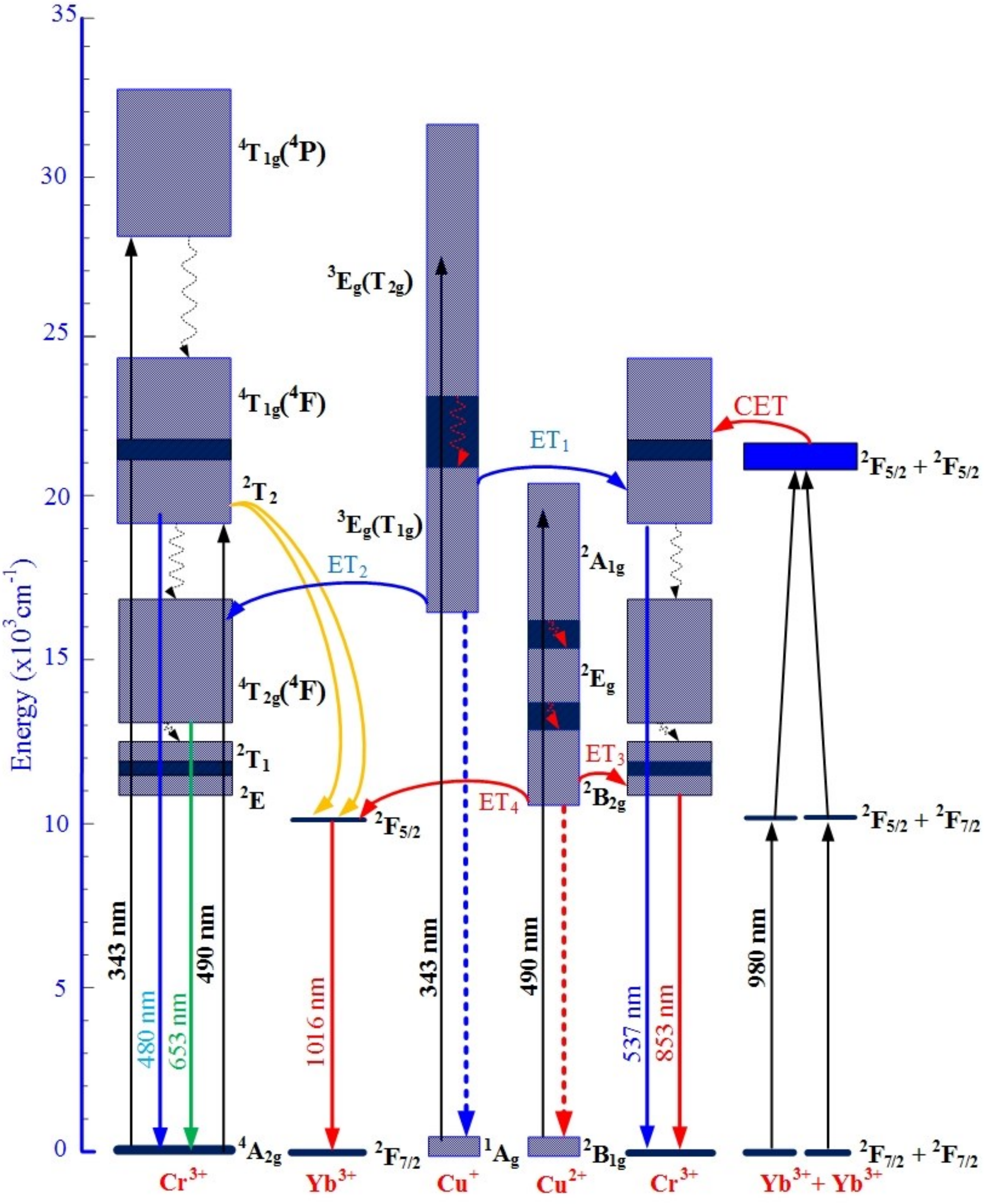

Fig. S10 Mechanism ET processes for the visible, UC, and NIR luminescence of Cr^{3+}/Yb^{3+} co-doped under excitations 343, 490, and 980 nm LD.

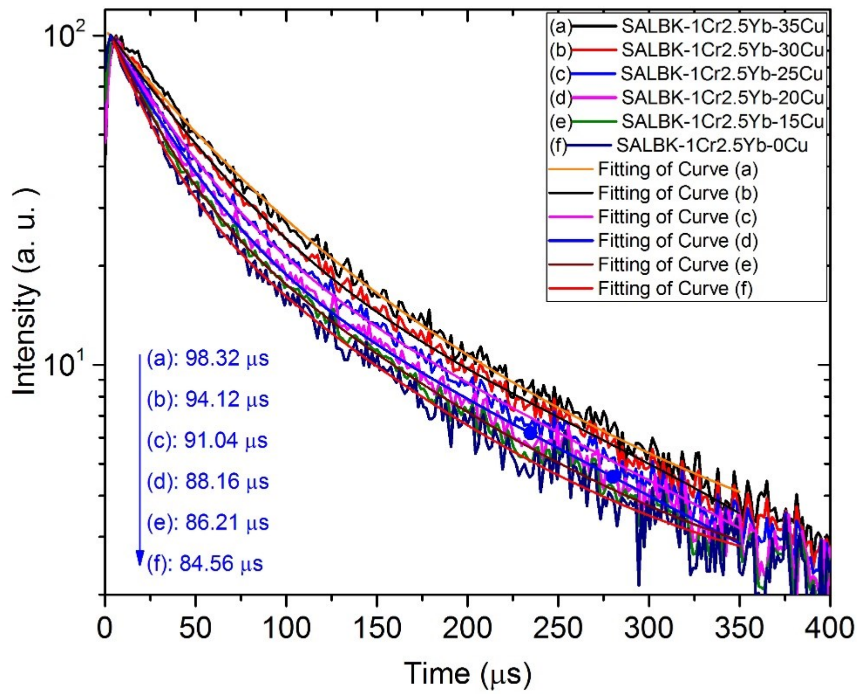

Fig. S11a Decay lifetimes curves of Cr^{3+} at 537 nm in SALBK-0Cu, SALBK-15Cu, SALBK-20Cu, SALBK-25Cu, SALBK-30Cu, and SALBK-35Cu glass samples, under excitation 980 nm.

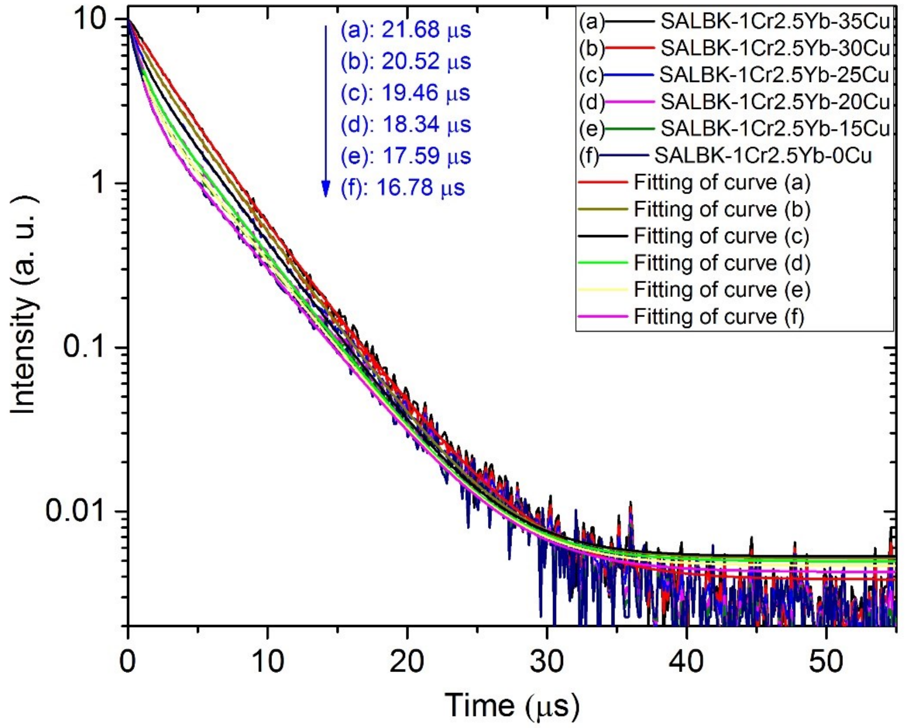

Fig. S11b Decay lifetimes curves of Cr^{3+} at 653 nm in SALBK-0Cu, SALBK-15Cu, SALBK-20Cu, SALBK-25Cu, SALBK-30Cu, and SALBK-35Cu glass samples, under excitation 343 nm.

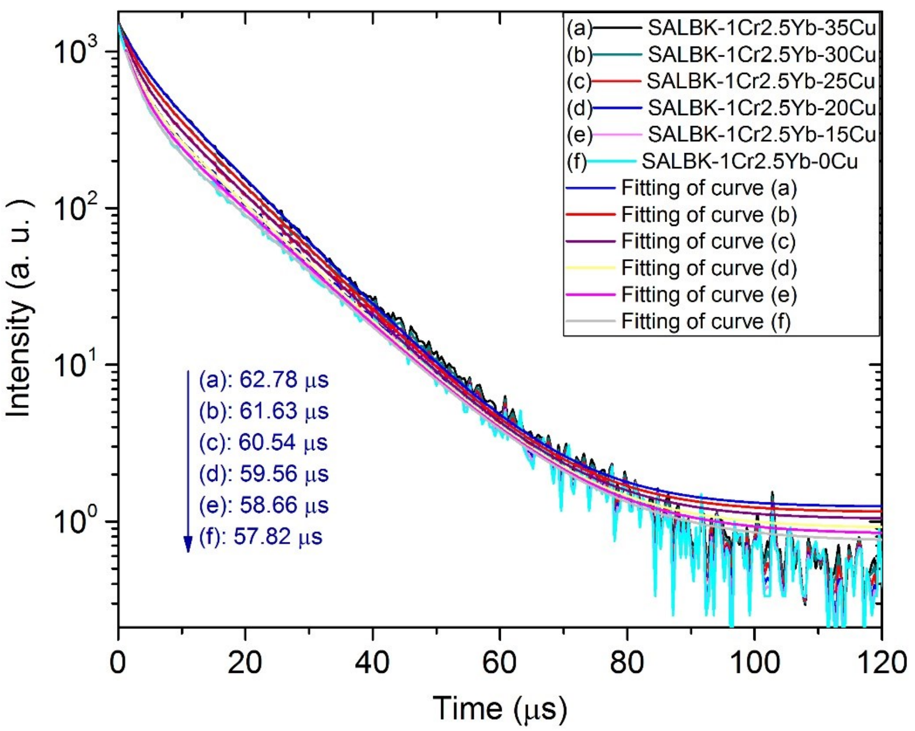

Fig. S11c Decay lifetimes curves of Cr^{3+} at 853 nm in SALBK-0Cu, SALBK-15Cu, SALBK-20Cu, SALBK-25Cu, SALBK-30Cu, and SALBK-35Cu glass samples, under excitation 980 nm LD.

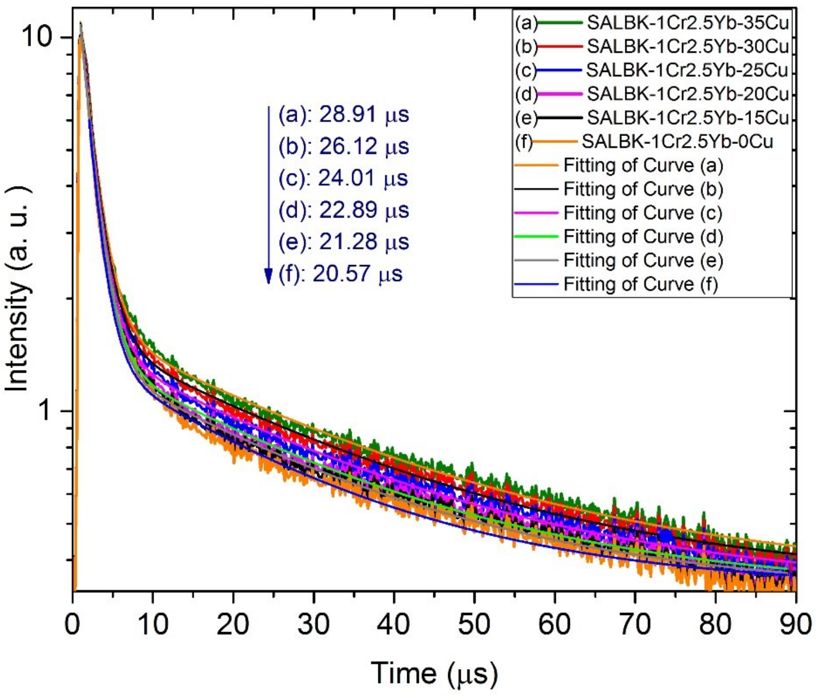

Fig. S11d Decay lifetimes curves of Yb^{3+} at 1016 nm in SALBK-0Cu, SALBK-15Cu, SALBK-20Cu, SALBK-25Cu, SALBK-30Cu, and SALBK-35Cu glass samples, under excitation 490 nm.

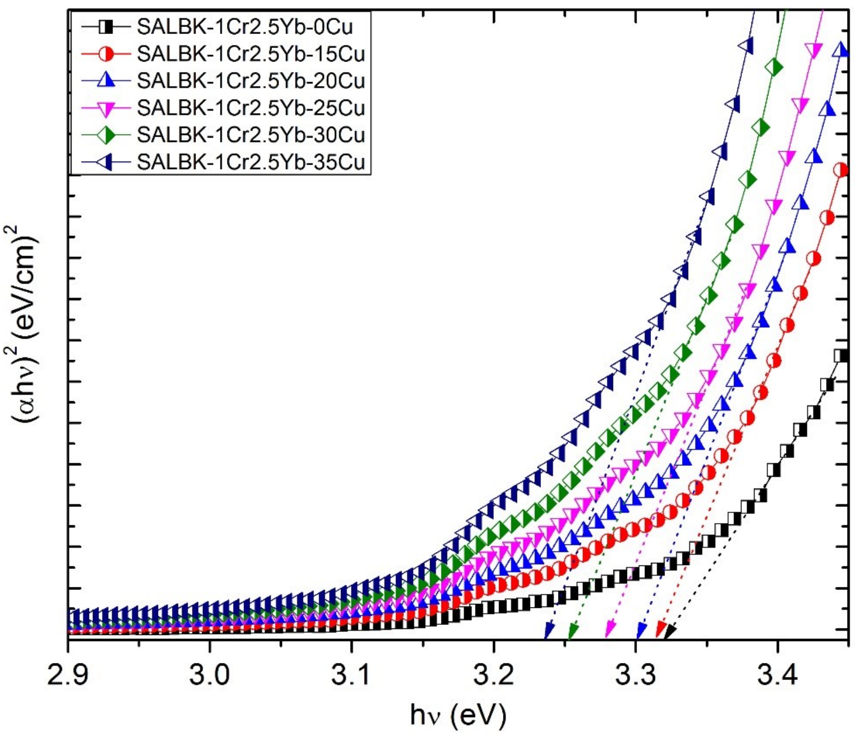

Fig. S12 Plot of (hν) versus $(\alpha h\nu)^2$ for estimating the E_g of SALBK-1Cr2.5Yb-0Cu, SALBK-1Cr2.5Yb-15Cu, SALBK-1Cr2.5Yb-20Cu, SALBK-1Cr2.5Yb-25Cu, SALBK-1Cr2.5Yb-30Cu, and SALBK-1Cr2.5Yb-35Cu glass samples.

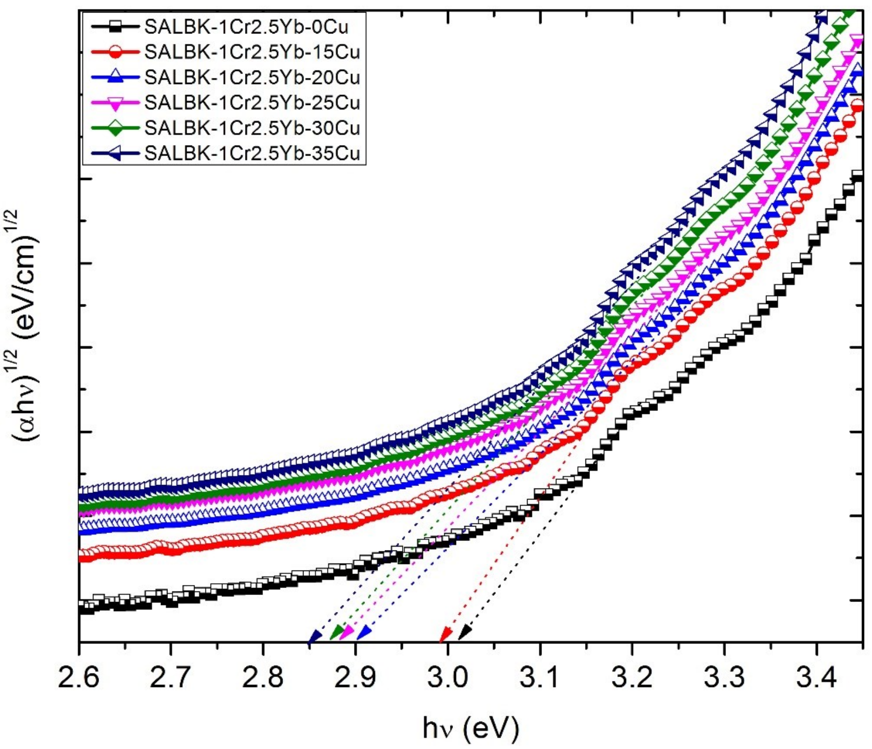

Fig. S13 Plot of (hν) versus $(\alpha h\nu)^{1/2}$ for estimating the E_g of SALBK-1Cr2.5Yb-0Cu, SALBK-1Cr2.5Yb-15Cu, SALBK-1Cr2.5Yb-20Cu, SALBK-1Cr2.5Yb-25Cu, SALBK-1Cr2.5Yb-30Cu, and SALBK-1Cr2.5Yb-35Cu glass samples.
